# Life satisfaction and positive and negative feelings of workers: a systematic review protocol

**DOI:** 10.1186/s13643-018-0903-6

**Published:** 2018-12-23

**Authors:** Viviane Cruvinel Di Castro, Janete Capel Hernandes, Mauro Elias Mendonça, Celmo Celeno Porto

**Affiliations:** Faculdade de Medicina. Programa Ciências da Saúde, Universidade Federal de Goiás, Secretaria – 1a s/n – Setor Universitário, CEP 74.605-020, Goiânia, Goiás Brazil

**Keywords:** Subjective well-being, Worker, Systematic review, Protocol

## Abstract

**Background:**

In this article, conceptualization of well-being is a starting point. According to Diener, subjective well-being refers to all kinds of evaluation, both positive and negative, people make about their own lives. It includes cognitive assessments, such as satisfaction with life and satisfaction with work, as well as affective reactions to life events, such as sadness and contentment. Low levels of health and well-being in workers lead to many consequences. Sick leave, low productivity, and absenteeism are some examples. In this systematic review, the main objective is to assess workers’ subjective well-being.

**Methods:**

The studies should include workers, whether they are paid or volunteers. Also, they must assess workers’ subjective well-being. Observational peer-reviewed studies will be included. Qualitative studies will be excluded. The primary outcomes to be considered are the subjective well-being indicators described. Only studies that used six (6) instruments, developed by Diener, will be included. The instruments are Satisfaction With Life Scale (SWLS), Scale of Positive and Negative Experience (SPANE), Positive Thinking Scale (PTS), Flourishing Scale (FS), Comprehensive Inventory of Thriving (CIT), and Brief Inventory of Thriving (BIT). The studies will come from Scientific Electronic Library Online (SciELO), Biblioteca Virtual em Saúde (BVS), Portal da Coordenação de Aperfeiçoamento de Pessoal de Nível Superior (CAPES), MEDLINE, Embase, Web of Science, and Global Index Medicus databases. The studies must be written in Portuguese, English, or Spanish.

**Discussion:**

As far as we know, this is the first systematic review related specially to workers’ subjective well-being. We hope that this study contributes to the “well-being at work” discussion and also to the development of effective interventions, used outside and inside organizations, that could improve well-being scores and increase correlate variables scores such as general health, social relations, and quality of life.

**Systematic review registration:**

PROSPERO CRD42016039520

**Electronic supplementary material:**

The online version of this article (10.1186/s13643-018-0903-6) contains supplementary material, which is available to authorized users.

## Background

In this article, conceptualization of well-being is a starting point. The article published by Diener in 1984, under the title “Subjective Well-Being,” shows that components and measures of subjective well-being were subjects of research for more than a decade and were therefore undergoing changes, assessments, and evolutions [1].

Research on subjective well-being has increased significantly, with approximately 14,000 publications per year surrounding the topic. There are five main recent findings: multidimensionality of subjective well-being, circumstances influencing it, cultural differences, health and social relation benefits, and interventions to increase subjective well-being [2].

According to Diener, subjective well-being refers to all kinds of evaluation, both positive and negative, people make about their own lives. It includes cognitive assessments, such as satisfaction with life and satisfaction with work, as well as affective reactions to life events, such as sadness and contentment. Thereby, subjective well-being can be seen under two distinctive angles: one affective or emotional, represented by positive and negative feelings, and other cognitive which corresponds to life satisfaction [3]. This concept can be applied in any condition, including workers.

According to Danna and Griffin [4], low levels of health and well-being in workers lead to many consequences. Sick leave, low productivity, and absenteeism are some examples and are part of the routine of public and private companies. Corporate employee assistance programs and major lifestyle changes are some of the interventions that can promote improvements in the health and well-being of the people who work.

The concept of subjective well-being published by Diener will be fundamental in the accomplishment of this systematic review. As pointed out in one review of self-report well-being measures [5], Diener’s subjective well-being model is among the most referenced theories in well-being studies.

A rationale for this review is to provide evidence that shows that Diener’s tools are preferable on the evaluation of subjective well-being when compared to other tools, especially because of their psychometric properties [6].

Given the diversity of concepts and scientific theories of subjective well-being, the authors decided to use the concept assumed by Diener and the instruments developed by him. Thus, the authors of the protocol assume only one concept and can solve a possible problem of the systematic review related to the impossibility of comparison and analysis of included studies due to the diversity of instruments and associated concepts.

Another essential term and not less important is the word worker. Thus, the present systematic review will aim to assess the subjective well-being of workers based on published research on the subject.

It is known that there are systematic reviews, concluded or ongoing, dedicated to study subjective well-being and its relation to non-labor factors such as personality traits, leisure, physical exercises [7–15], and labor factors such as income and job satisfaction [16, 17]. However, until now, the authors of this protocol are unaware of any systematic review that addresses the subjective well-being of workers. Therefore, this will be the first systematic review devoted to this topic, and it will also be an opportunity to evaluate the quality of primary studies conducted on workers’ well-being.

### Objectives

We will perform a systematic review of studies exploring subjective well-being of workers. The study aims to answer the following research questions: (1) What is the level of workers’ subjective well-being? (2) Are there differences between different types of workers?

Thereby, and more specifically, the objectives of this systematic review are:

1. Assess life satisfaction and positive and negative feelings of workers
2. Compare subjective well-being (life satisfaction and feelings) of workers of different professional categories, if applicable

## Methods

The review was recorded in PROSPERO [18] database (registration number CRD42016039520). This protocol was structured according to the Preferred Reporting Items for Systematic Review and Meta-Analysis Protocols (PRISMA-P) [19] guidance [see Additional file 1].

### Eligibility criteria

Studies will be selected according to the criteria outlined below.

#### Participants

The studies should include workers, whether they are paid or voluntary workers. In order to be qualified as a worker, participants must have a formal relationship with the company where they work (contract, worker’s number), regardless of their occupational category, and they must be employed at the time the studies were performed. Retirees will be excluded. Also, the studies must assess workers’ subjective well-being.

#### Study designs

Observational peer-reviewed studies will be included. Gray literature (such as theses, dissertations, books, and papers presented in congresses) will also be considered. Qualitative studies will be excluded. The authors of the protocol decided that the qualitative studies found in the searches will be used in a second study.

#### Interventions or exposure

Not applicable.

#### Comparators

A comparison will be made between different types of workers based on the International Standard Classification of Occupations (ISCO) [20], if applicable.

#### Primary outcomes

The primary outcomes to be considered are the subjective well-being indicators described in the studies.

Since the number of instruments to evaluate such a construct is very broad, and we will consider the concept of subjective well-being described by Diener in this systematic review, only studies that used six (6) instruments, also developed by Diener to evaluate subjective well-being, will be included. They are Satisfaction With Life Scale (SWLS) [21], Scale of Positive and Negative Experience (SPANE) [6], Flourishing Scale (FS) [6], Positive Thinking Scale (PTS) [22], Comprehensive Inventory of Thriving (CIT) [23], and Brief Inventory of Thriving (BIT) [23].

If the study has more than one variable being evaluated, only those studies that provide “subjective well-being” results separately will be considered for the purpose of this review.

We intend to divide the studies into groups for analysis purposes. Comparisons will be made between studies using the same tool to measure the same outcome. For example, a study that used SWLS to evaluate life satisfaction will have its results compared to other studies that have also used SWLS to evaluate life satisfaction. Together, such studies will be considered a group and the comparison will be made in this specific variable level.

#### Secondary outcomes

Secondary outcomes will be not considered in this review.

### Search methods

Literature search strategies will be developed using Medical Subject Headings (MeSH), Descritores em Ciências da Saúde (DeCS), Embase Thesaurus (Emtree), and text words. We will search MEDLINE [24], Scientific Electronic Library Online (SciELO) [25], Biblioteca Virtual em Saúde (BVS) [26] database, Coordenação de Aperfeiçoamento de Pessoal de Nível Superior – CAPES [27] data, Embase [28], Web of Science [29], and Global Index Medicus [30]. The data will be reached from inception until December 15, 2018.

To ensure literature saturation, we will scan the reference lists of included studies or relevant reviews identified through the search. For searches in the databases, the following keywords or search terms will be used: “subjective well-being,” “worker,” “work,” “occupation,” “employee,” “volunteer,” “voluntary workers,” “satisfaction with life,” and “positive and negative feelings.”

The data found in the search process requires the use of EndNote X9 for this process step. If repeated studies are identified, they will be removed from the research. Only studies in Portuguese, English, and Spanish will be accepted. An example of the final research strategy used for the MEDLINE database is provided [see Additional file 2]. A search in four databases indicating initial results can be seen in Table 1.

**Table 1:** Number of studies per base

Database	Results
Medline	11,774
BVS	1551
SciELO	81
CAPES	506,068
Total	519,474

*SciELO* Scientific Electronic Library Online, *BVS* Biblioteca Virtual em Saúde, *CAPES* Coordenação de Aperfeiçoamento de Pessoal de Nível Superior

### Selection of studies

Two reviewers will independently screen the studies identified by the searches following a three-phase procedure. After each phase, the reviewers will check inclusions and exclusions, and in case of disagreements, a third reviewer will be involved as an adjudicator.

For phase 1, titles of the articles identified by the searches will be screened. If repeated studies are identified, they will be removed from the research.

For phase 2, abstracts of articles will be screened against the following criteria:

1. Is the study related to subjective well-being? (yes, not clear, or no)
2. Is the study with workers? (yes, not clear, or no)

For phase 3, full texts of the studies selected on phase 2 will be read and screened against the following criteria:

1. Is it a quantitative study? (yes, not clear, or no)
2. Is the study in Portuguese, English, or Spanish? (yes, not clear, or no)
3. Did the study use any instrument developed by Diener to evaluate participants’ subjective well-being? (yes, not clear, or no)
4. Did the study provide “subjective well-being” results separately? (yes, not clear, or no)

After that, data of the studies meeting all the above inclusion criteria will be used to elaborate a table with the following information:

1. Authors’ names
2. Date of the study publication
3. Country of origin
4. Study design
5. Summary of studies containing the following information: objective, sample size, type of work (professional categories, voluntary work, non-voluntary work), well-being variables, instruments, results, and conclusions

Reasons for the studies exclusion will be recorded. None of the review authors will be blind to the journal titles, authors, or study institutions. A flow diagram of the study will be done containing measures, such as identification, screening, eligibility, and inclusion and an explanatory statement on the grounds of exclusion.

### Data extraction and management

Reviewers will extract data independently from each eligible study. Data abstracted will include demographic information, variables, and outcomes.

Age and gender are examples of demographic variables. Both will be extracted in mean and proportion values, respectively.

In order to avoid overlapping reports, multiple reports of a single study will be identified and excluded.

### Quality assessment

To assess the risk of bias in studies, JBI Critical Appraisal Checklist tools, presented by International Joanna Briggs Institute in 2017, will be used [31].

If the studies are evaluated with an indication of high risk of bias in more than half of the items proposed by the tool, it will be considered a study with high risk of bias. If the studies are evaluated with indication of high risk of bias in half or less than half of the items of the tool, it will be considered with medium or low risk of bias.

Grading of Recommendations Assessment, Development and Evaluation (GRADE) [32] approach will be used for describing the quality of relevant evidence, if applicable.

### Evidence synthesis

A systematic narrative synthesis will be provided with information presented in the text and tables to summarize and explain the characteristics and well-being results of the included studies. The narrative synthesis will explore the relationship and findings both within and between the included studies.

If there is a very high heterogeneity between studies, metanalysis will not be performed. High heterogeneity will be determined on the basis of type of work, age of participants, proportion of males/females, and type of organization. In case of homogeneity, metanalysis will be conducted.

For metanalysis, heterogeneity will be assessed using the *I*^2^ based on the Q statistic of Cochran test. To determine the level of heterogeneity, Cochrane classification for *I*^2^ values will be used. If *I*^2^ is 50% or less, there is little heterogeneity. If *I*^2^ value is between 51 and 75%, there is medium heterogeneity. If *I*^2^ is above 75%, there is high heterogeneity between the included studies [33].

Heterogeneity will be explained by subgroup analysis using the following variables: age, gender, study design, study language, instruments used to evaluate subjective well-being, type of work, type of organization, risk of study bias, and method of analysis of the results.

Outcomes will be analyzed using raw mean differences. The results will be presented on a forest plot.

Metanalyses will be conducted by using random-effects method (DerSimonian and Laird), because confidence intervals for the average intervention effect will be wider and corresponding claims of statistical significance will be more conservative [34–36].

Three different tools will be used to assess meta-biases such as publication bias and outcome reporting bias. If 10 or more studies are available, the potential for publication bias will be explored through funnel plots. Additionally, Begg and Mazumdar’s test [37] and Egger’s test [38] will be used to assess small study effects. Also, according to the tests, if *p* < 0.05, publication bias will be detected. If *p* ≥ 0.05, there will be no publication bias.

Finally, for completing metanalyses, RevMan 5.3 will be used [39].

### Amendments to protocol

Any substantive amendments to this protocol will be registered with PROSPERO as they occur and documented in the final publication.

### Dissemination

We will publish review results in an international peer-reviewed journal and will report results according to the Preferred Reporting Items for Systematic Reviews and Meta-Analyses (PRISMA) statement.

We will also disseminate results to the research community and relevant key stakeholders through presentations at relevant academic and non-academic meetings and via social media. If findings are found to be interesting to the wider public, we will disseminate them via mass media.

## Discussion

As far as we know, this is the first systematic review related specially to workers’ subjective well-being. The evidence synthesis of the review will probably aid in the comparison of well-being results. We hope that this study contributes to the “well-being at work” discussion and also to the development of effective interventions, used outside and inside organizations, that could improve well-being scores and increase correlate variables scores such as general health, social relations, and quality of life.

In addition, the research will be part of a doctoral thesis, articles, posters, and discussions which may instigate further discussions on the subject in the academic community and, consequently, the preparation of proposals and guidelines designed to enhance subjective well-being of workers in general.

This systematic review may present some potential limitations. One of them may be the fact that we will not include studies that were presented at scientific events and have not been published in scientific journals. Also, studies in languages other than English, Portuguese, and Spanish will not be included.

At the time of data analysis, we may have problems with the lack of homogeneity of the studies. This has been a difficulty for reviewers when undertaking systematic reviews of the literature. The challenge of drawing conclusions based on very diverse studies and samples is enormous and may hamper the generalization of results.

## Additional files

Additional file 1: Preferred Reporting Items for Systematic Reviews and Meta-Analysis Protocols (PRISMA-P) 2015 checklist. (DOC 86 kb)

Additional file 2: Search strategies MEDLINE. Medline search strategy. (DOCX 13 kb)

## Abbreviations

BIT: Brief Inventory of Thriving
BVS: Biblioteca Virtual emSaúde
CAPES: Coordenação de Aperfeiçoamento de Pessoal de Nível Superior
CI: Confidence intervals
CIT: Comprehensive Inventory of Thriving
DeCS: Descritores em Ciências da Saúde
Emtree: Embase Thesaurus
FS: Flourishing Scale
ISCO: International Standard Classification of Occupations
MeSH: Medical Subject Headings
PRISMA: Preferred Reporting Items for Systematic Reviews and Meta-Analyses
PRISMA-P: Preferred Reporting Items for Systematic Review and Meta-Analysis Protocols
PTS: Positive Thinking Scale
SciELO: Scientific Electronic Library Online
SPANE: Scale of Positive and Negative Experience
SWLS: Satisfaction With Life Scale
