# Supplementary material for: Life satisfaction and positive and negative feelings of workers: a systematic review protocol
Source: Syst Rev. 2018 Dec 23;7:243. doi: 10.1186/s13643-018-0903-6 (PMC6304232; doi:10.1186/s13643-018-0903-6)
Supplement: Supplementary file 2 — Search strategies MEDLINE. Medline search strategy. (DOCX 13 kb) [file 13643_2018_903_MOESM2_ESM.docx]

**Additional file 2 : Example of search stratgey in MedLine**

subjective[All Fields] AND ("health"[MeSH Terms] OR "health"[All Fields] OR "well"[All Fields] OR "well being"[All Fields]) OR ("life"[MeSH Terms] OR "life"[All Fields]) AND ("personal satisfaction"[MeSH Terms] OR ("personal"[All Fields] AND "satisfaction"[All Fields]) OR "personal satisfaction"[All Fields] OR "satisfaction"[All Fields]) OR ("personal satisfaction"[MeSH Terms] OR ("personal"[All Fields] AND "satisfaction"[All Fields]) OR "personal satisfaction"[All Fields] OR "satisfaction"[All Fields]) OR positive[All Fields] AND ("emotions"[MeSH Terms] OR "emotions"[All Fields] OR "feelings"[All Fields]) OR negative[All Fields] AND ("emotions"[MeSH Terms] OR "emotions"[All Fields] OR "feelings"[All Fields]) OR ("emotions"[MeSH Terms] OR "emotions"[All Fields] OR "feelings"[All Fields]) AND ("manpower"[Subheading] OR "manpower"[All Fields] OR "workers"[All Fields]) OR "work"[MeSH Terms] OR "work"[All Fields] OR "occupations"[MeSH Terms] OR "occupations"[All Fields] OR "occupation"[All Fields] OR employee[All Fields] OR "volunteers"[MeSH Terms] OR "volunteers"[All Fields] OR "voluntary"[All Fields] OR "voluntary workers"[All Fields]
